# ^18^F-deoxyglucose positron emission tomography/computed tomography to predict local failure in esophageal squamous cell carcinoma

**DOI:** 10.18632/oncotarget.15606

**Published:** 2017-02-22

**Authors:** Bingjie Fan, Pingping Fan, Li Kong, Xindong Sun, Shuqiang Zhao, Xiaorong Sun, Zheng Fu, Jinsong Zheng, Li Ma, Shijiang Wang, Man Hu, Jinming Yu

**Affiliations:** ^1^ Shandong Cancer Hospital Affiliated to Shandong University, Jinan, China; ^2^ Shandong Academy of Medical Sciences, Jinan, China; ^3^ Departments of Radiation Oncology and Shandong Province Key Laboratory of Radiation Oncology, Shandong Cancer Hospital and Institute, Jinan, China; ^4^ Departments of Nuclear Medicine, Shandong Cancer Hospital and Institute, Jinan, China

**Keywords:** esophageal squamous cell carcinoma, radiotherapy, FDG PET/CT, concurrent chemoradiotherapy, local failure

## Abstract

Esophageal squamous cell carcinoma (ESCC) patients are at risk for local failure (LF) following treatment. Predicting tumor regions at high risk for local failure before radiotherapy may increase treatment efficacy by permitting an escalated radiation dose specifically to those regions critical for tumor control. Forty-one patients with non-resectable locally advanced ESCC underwent ^18^F-deoxyglucose positron emission tomography/computed tomography (FDG PET/CT) imaging before concurrent chemoradiotherapy (CCRT). After CCRT, a second (failure) FDG PET/CT was performed in cases of relapse. Failure FDG PET/CT scans were fused to pre-treatment scans to identify tumor regions at high risk for LF. Within a median follow-up time of 26 months, 20 patients (48.8%) had LF. In 19 patients, the failure occurred within a pre-treatment high FDG uptake region; the failure occurred outside these regions in only one patient. Pre-treatment metabolic tumor volume (MTV) was independently associated with LF (*P*<0.001, HR 1.128, 95% CI: 1.061–1.198). LF was more likely in patients with MTVs ≥27 cm^3^. In initial PET/CT images, when 50% maximum standardized uptake value (SUV_max_) was used as the threshold, delineated subvolumes overlapped LF regions. These results confirm that LF occurs most commonly within pre-treatment high FDG uptake regions.

## INTRODUCTION

Esophageal cancer (EC) is the eighth most common cancer and the sixth leading cause of cancer-related mortality worldwide [1]. More than 50% of patients with EC are diagnosed at late stages and tumors are not amenable to surgery [2]. Concurrent chemoradiotherapy (CCRT) is the standard treatment for locally advanced inoperable EC cases, as per Radiation Therapy Oncology Group (RTOG) phase III intergroup trial results (85-01) showing improved local control (LC) and overall survival (OS) with CCRT compared with radiotherapy (RT) alone [3, 4]. Recently, definitive chemoradiotherapy showed the potential to achieve the same survival benefit as surgery in locally advanced EC [5, 6]. Despite advances in chemotherapy and RT, local failure (LF) is still observed in nearly half of patients with locally advanced EC treated with CCRT [4, 7], and LF is associated with poor OS [4, 8, 9].

It is currently challenging to identify tumor regions at high risk for LF. The most common strategy for improving LC of EC is escalating the radiation dose. However, an RTOG phase III trial (94-05) showed that escalating the dose to 64.8 Gy did not improve local-regional control or survival [7], and treatment time was prolonged due to toxicity in high-dose arms. Notably, RT planning in this trial was based on conventional imaging modalities.

^18^F-deoxyglucose positron emission tomography/computed tomography (FDG PET/CT) provides additional information on the pathophysiological and biological characteristics of a tumor [10, 11], and may better assess tumor radio-resistance [12, 13]. In a pre-clinical model, radiation dose escalation showed better LC for tumors with higher FDG uptake compared to those with lower uptake [14]. This suggested that at the tumor level, high FDG uptake zones might exhibit residual metabolic activity and increased risk for LF [14]. Therefore, FDG PET/CT-based definition of high-risk tumor sub-volumes may increase RT efficacy by permitting an escalated dose to regions critical for disease control. Studies investigating relationships between high FDG uptake regions and treatment failure have been performed in solid tumors, such as non-small cell lung cancer (NSCLC) [12, 15–19], head and neck cancer [20–22], pancreatic cancer [23], and rectal cancer [24]. The high FDG uptake regions were suggested to be responsible for LF [12, 15–24]. To the best of our knowledge, high tumor FDG uptake before CCRT has not yet been employed to identify regions at high risk for LF in ESCC.

The present study explored tumor regions at high-risk for LF after CCRT in ESCC using FDG PET/CT. We hypothesized that high FDG uptake areas would be more prone to LF. We also assessed FDG PET/CT-related characteristics and other clinical factors for their potential application as risk factors for identifying LF in these regions.

## RESULTS

### Baseline patient and tumor characteristics

Forty-one patients were included in this study. Median patient age was 58 years (range, 26–78 years); there were 32 (78%) men and nine (22%) women. Endoscopic ultrasound examination was limited in some patients due to esophageal obstruction. It was difficult to differentiate T2 from T3 lesions, so we divided patients into stage T1–3 vs. T4. Patient and tumor characteristics are shown in Table 1. All patients had abnormal FDG uptake before treatment. The mean initial PET/CT maximum standard uptake value (SUV_max_) and metabolic tumor volume (MTV) were 11.07±3.35 (range, 4.73–16.70) and 27.72±11.08 cm^3^ (range, 11.35–47.20 cm^3^), respectively.

**Table 1:** Baseline data regarding patient and tumor characteristics

Characteristics	Value	Percentage
Age, years		
Median	58	
Range	26 - 78	
Sex		
Female	32	78.0
Male	9	22.0
Tumor length, cm		
Median	5	
Range	3.0 - 8.5	
T category		
T1-3	34	82.9
T4	7	17.1
Lymph node category		
N0	9	22.0
N1-3	32	78.0
Tumor location		
Cervical	6	14.6
Upper thoracic	13	31.7
Mid-thoracic	15	36.6
Lower thoracic	7	17.1
Pre-PET/CT		
	Mean ± SD	Range
SUV_max_	11.07 ± 3.35	4.73 - 16.70
MTV, cm^3^	27.72 ± 11.08	11.35 - 47.20

*Abbreviations*: SUV_max_: maximal standardized uptake value; MTV: metabolic tumor volume.

### Survival and LF

**Figure 1:**
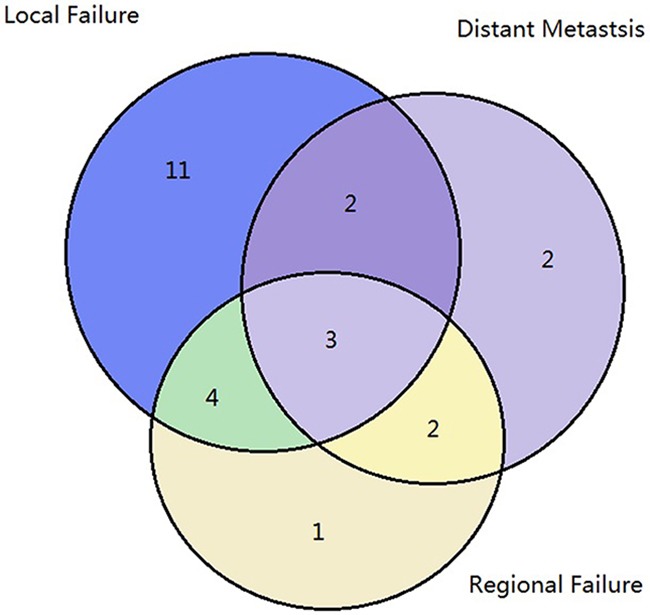
Failure patterns Patterns of failures are shown based on the first treatment failure; 48.8% of the patients had local failure during the observation period and 12.2% experienced failure without primary tumor.

Median follow-up time was 26 months (range, 8–42 months), and 2-year OS was 53.5%. Details of the first failure event are presented in Figure 1. Twenty patients (48.8%) had LF during the observation period. Eleven (26.8%) of these had failure in the primary tumor area while nine (22.0%) had failure in the regional lymph nodes and/or distant sites. Five (12.2%) patients experienced failure without primary tumor area involvement. Sixteen (39.0%) patients had no evidence of disease at the last follow-up. The median LF time was 11.1 months (range, 5.4–19 months). The 2-year LC was 51.2%. All LFs occurred during the first two years following treatment, with 11 patients in the first year and 9 in the second.

### Factors associated with LF

LC was associated with tumor T/N staging and size. The 2-year LC was 55.4% for patients with stage T1–3 tumors and 28.6% for patients with stage T4 tumors (*P*=0.026); 72.9% for N0 patients and 16.7% for patients with positive lymph nodes (*P*=0.032); and 71.2% for patients with tumors <5 cm and 22.5% for patients with tumors ≥5 cm (*P*<0.001). In univariate COX regression analyses, tumor length (*P*=0.001), T stage (*P*=0.034), N stage (*P*=0.048), and initial PET/CT MTV (*P*=0.001) of the primary tumor were associated with LF (Table 2). Initial PET/CT MTV was an independent risk factor for LF in multivariate analyses (*P*<0.001, HR 1.128, 95% CI: 1.061–1.198), while T stage (*P*=0.182), N stage (*P*=0.053) and tumor length (*P*=0.135) were not.

**Table 2:** Univariate analysis for local control

Variable	Categories	HR	95% CI	*P*
Age	<60 versus ≥ 60	1.000	0.960-1.043	0.984
Sex	Men versus women	0.503	0.148-1.715	0.272
Location Cervical	Cervical versus other site	0.832	0.245-2.832	0.769
Location Upper thoracic	Upper thoracic versus other site	1.540	0.646-3.671	0.330
Location Mid-thoracic	Mid-thoracic versus other site	0.861	0.334-2.223	0.757
Location Lower thoracic	Lower thoracic versus other site	0.791	0.211-2.451	0.719
Tumor length	< 5 cm versus ≥ 5 cm	6.090	2.286-16.222	0.001
T category	T1-3 versus T4	2.846	1.081-7.488	0.034
N category	N0 versus N1-3	4.436	1.011-19.469	0.048
SUV_max_		0.975	0.850-1.118	0.719
MTV		1.128	1.061-1.198	0.001

*Abbreviations*: SUV_max_: maximal standardized uptake value; MTV: metabolic tumor volume.

### ROC analysis

**Figure 2:**
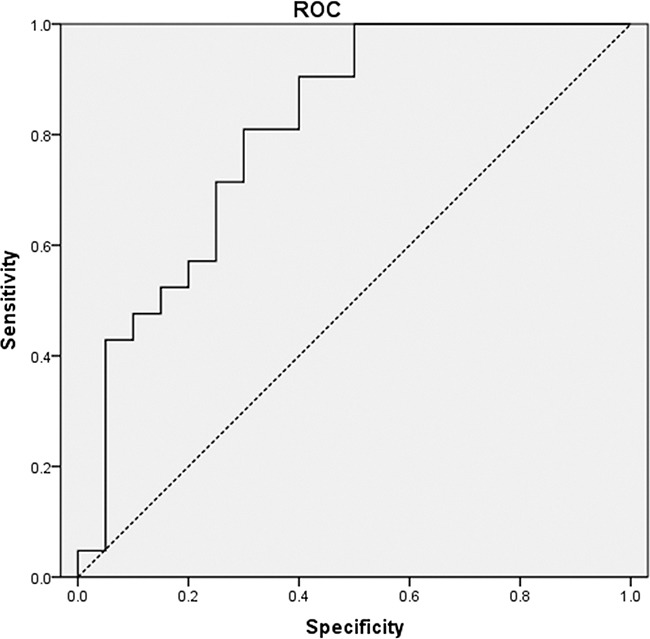
Receiver operating characteristics (ROC) analysis of metabolic tumor volume (MTV) ROC curve using pre-PET/CT MTV to predict local failure. The area under the curve (AUC) of pre-PET/CT MTV was 0.810 (*P*=0.001).

We evaluated the optimal cut-off value for tumor initial PET/CT MTV for predicting LF. AUC of initial PET/CT MTV was 0.810 (*P*=0.001; Figure 2). The optimal cut-off value for initial PET/CT MTV was 27 cm^3^.

### Spatial location relationship between initial PET/CT subvolumes and LF

**Figure 3:**
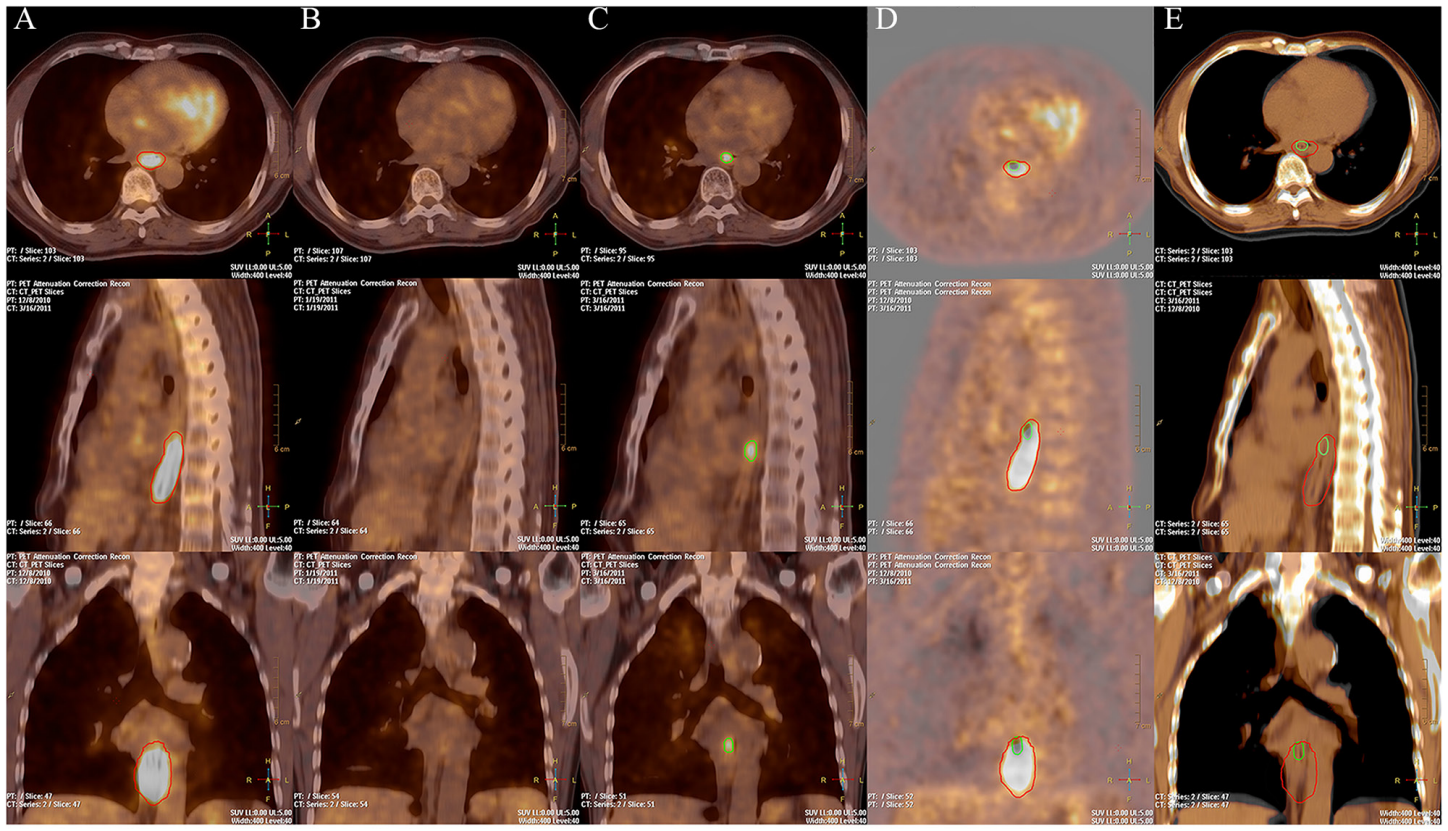
PET/CT images of a representative patient The pre-treatment **A**. post-treatment **B**. and local failure **C**. PET/CT images of a representative patient. The red lines indicate subvolumes with high FDG uptake in pre-treatment PET/CT. Green lines indicate local failure region in failure PET/CT. The fusion PET images **D**. and CT images **E**. of the pre-PET/CT and failure PET/CT shows a large correspondence between the local failure regions with the high FDG uptake areas pre-treatment.

**Figure 4:**
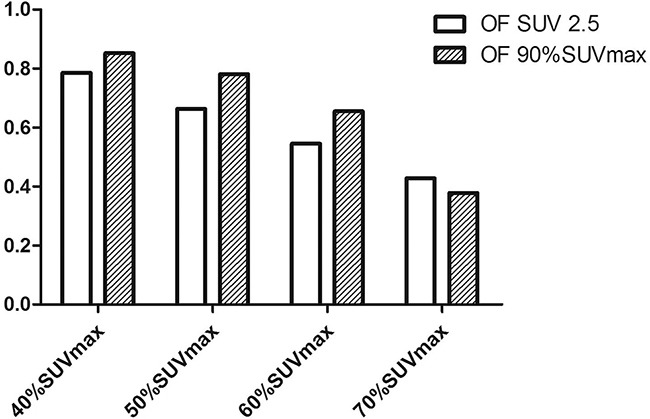
Overlap fraction (OF) for the hotspot after local failure for different maximal standardized uptake values (SUV_max_) The OF values for 40%SUV_max_, 50%SUV_max_, 60%SUV_max_, and 70%SUV_max_ were 85.2%, 78.1%, 65.5%, and 37.8%, respectively.

LF areas (with SUV threshold of 2.5) were transposed to the initial PET/CT scans to assess whether LF regions were located within the subvolumes defined by different thresholds. Among all patients with LF, only one (5%) had LF in an area that had no overlap with the initial PET/CT high uptake region. A representative patient with LF in initial PET/CT high FDG uptake regions is shown in Figure 3. For the 19 patients who had LF in initial PET/CT high uptake regions, the average OF of the failure regions with Pre40%, Pre50%, Pre60%, and Pre70% were 78.6%, 66.3%, 54.5%, and 42.8%, respectively. OF for the hotspot after LF and these pre-PET/CT subvolumes were 85.2%, 78.1%, 65.5%, and 37.8%, respectively (Figure 4).

## DISCUSSION

LF is a major concern for patients with ESCC. Identifying regions at high risk for LF could help tailor individual patient treatment approaches. This study assessed whether FDG PET/CT could predict LF risk regions in patients with non-resectable locally advanced ESCC. Results showed that LF in ESCC appears most commonly within high FDG uptake regions. We propose a 50% SUV_max_ threshold to define subvolumes that would benefit from dose escalation. Patients with MTV ≥27 cm^3^ are more likely to have failure in these regions, and in these cases, more effective strategies like dose escalation should be employed.

Treatment outcomes in locally advanced EC are still poor and nearly 50% of patients exhibit LF in the primary tumor or affected lymph nodes [8]. In this study, among patients treated with CCRT, 2-year OS was 53.5% and 2-year LC was 51.2%. LF is the most common failure pattern. The results of the present study were similar to those of Welsh, *et al*. [8]. The RTOG 94-05 trial failed to demonstrate a LC or survival advantage at higher RT doses. This result suggested that delivering a uniformly distributed dose to the heterogeneous tumor volume might be inappropriate. We hypothesized that escalating radiation doses to specific tumor areas more likely to experience a LF could improve tumor control without increasing radiation toxicity. Our results strongly suggest that tumor subvolumes identified as high FDG uptake areas via PET/CT are at high risk for LF in EC patients treated with CCRT.

Few studies have focused on identifying regions at high risk for LF in EC. Versteijne, *et al*. [27] showed that most LF occurs at the primary tumor site (86%). Button, *et al*. [28] found that local relapse mostly occurred in the primary tumor area within RT fields. Welsh, *et al*. [8] found that LFs after CCRT often occur within the GTV. Unfortunately, pre-treatment PET/CT data, which may contain information about tumor biology, was unavailable in these studies. As we know, conventional imaging modalities only provide limited anatomic information based on the electronic densities of various tissues, but FDG PET/CT could be used to predict radiotherapy resistance [14, 27, 28]. In the present study, comparing FDG PET/CT data suggested that LF most commonly (95.2%) occurred in the pre-treatment high uptake regions. This result supports the hypothesis that areas of increased FDG uptake have some degree of resistance to RT. One patient (4.8%) experienced LF in the esophagus outside of the high FDG uptake regions. The most likely explanation for this is EC intramural skip metastasis. Still, we suggest that high uptake regions that make up the bulk of the tumor burden are more likely to persist or recur after CCRT.

In the present study, the Pre40% and Pre50% volumes had high overlap (78.6% and 66.3%, respectively) with LF regions, only covering an average of 50.6% and 37.5% of the pre-PET/CT MTV, respectively. These subvolumes also had high overlap with LF hotspots. In addition, both volumes overlapped the failure sites, but the Pre50% volume was smaller, which could lead to reduced radiation toxicity. Therefore, 50% SUV_max_ appears to be an acceptable threshold for dose escalation. These results are supported by the work of Calais, *et al*. [29], who observed that high FDG uptake regions on initial PET/CT scans identified tumor subvolumes at greater risk of recurrence after RT in patients with locally advanced EC [29]. In addition, a threshold of 60% of SUV_max_ appeared to be an acceptable choice for dose escalation [29].

The present study explored risk factors for ESCC LF after CCRT. The findings suggest that among ESCC patients treated with CCRT, those who initially presented with a PET/CT MTV >27 cm^3^ were at higher risk for LF. For these patients, more effective strategies may be needed to improve LC and survival, including new chemotherapeutics [30] and RT dose escalation. As SUV_max_ correlates with tumor activity and size, we would expect tumors with greater SUV_max_ to be more resistant to local treatment, and several studies demonstrated that SUV_max_ is a predictor for EC recurrence [31, 32]. However, in the present study, SUV_max_ was not associated with failure in high uptake regions. One possible reason for this discrepancy is that SUV_max_ does not necessarily represent tumor activity for the whole tumor mass, because a single pixel may not be representative of non-homogeneous overall tumor uptake. Instead, MTV, which combined tumor volume and total tumor metabolic activity, may reflect the metabolic burden of the active tumor more accurately.

Similar studies have been performed in NSCLC [12, 15–19], head and neck cancers [20–22], rectal cancers [24] and pancreatic cancer [23]. Aerts, *et al*. [18] confirmed that the locations of low and high FDG uptake areas within the tumor remained stable during radiotherapy. Because it is difficult to distinguish between radiation esophagitis and esophagus tumors via FDG PET/CT, this examination is not normally performed during RT at our center. Shusharina, *et al*. [15] performed four serial FDG PET/CT examinations after therapy, and found that OF becomes smaller as recurrent tumors grow larger. The greatest overlap was observed in early PET/CT scans and decreased in subsequent scans. Recurrent tumor growth locally into adjacent lung tissue and bronchus depends on many factors, including blood supply, and is expected to be non-uniform. Therefore, the best time to evaluate a failure location in the esophagus using FDG PET/CT remains unclear. Our main study limitation was the relatively low number of included patients. Further research with larger patient numbers and other indexes to identify regions for dose painting are needed to validate our conclusions.

In conclusion, LF in ESCC patients most commonly occurred in high FDG uptake regions after CCRT. Pre-treatment MTV was an independent risk factor for failure in high uptake regions, and a cut-off value of 27 cm^3^ predicted failure in these regions. Subvolumes defined by a threshold of 50% SUV_max_ may be an appropriate target volume for escalated radiation dose. For patients at high risk for LF, more aggressive treatments, such as higher radiation doses or combination chemotherapy, should be explored.

## MATERIALS AND METHODS

### Study design and patients

This study included patients with ESCC treated in our institute between June 2010 and February 2012. Ethical clearance for the study was obtained from the institutional review board and the ethics committees of Shandong Cancer Hospital. All patients gave written informed consent prior to enrolling in the study. Inclusion criteria were: 1) histologically diagnosed ESCC; 2) good performance status (Eastern Cooperative Oncology Group (ECOG) score ≤1); 3) adequate hematologic, hepatic, and renal function for CCRT; and 4) locally advanced disease, comorbidities, or patient's will making the tumor ineligible for surgery. Exclusion criteria were: 1) distant metastasis or multiple primary esophageal lesions; 2) other malignancies; or 3) history of chest RT, systemic chemotherapy, targeted therapy, or esophageal surgery. Of 45 patients screened, three were lost before follow-up was completed, and one discontinued treatment at an early stage; 41 patients met the inclusion criteria and were evaluable in the final analysis.

### Treatment

All patients were treated with CCRT. An initial CT scan with the patient in the treatment position was performed for simulation and to inform treatment planning. Radiation treatments were delivered as three-dimensional conformal RT (3D-CRT) or intensity modulation RT (IMRT). Treatment plans were generated using the Pinnacle planning system (ADAC-Pinnacle 3, version 5.0; Philips, Best, The Netherlands). Gross tumor volume (GTV), including the primary tumor and metastatic lymph nodes, was contoured on the planning CT scans using all available resources, including data from pre-treatment FDG PET/CT (pre-PET/CT) fusion scans, but without identifying specific RT boosting areas. Clinical tumor volume (CTV) was defined as GTV plus a 3.0-cm margin superior and inferior to the primary tumor, and a 1.0-cm radial margin plus the regional draining lymphatics, depending on the primary tumor location. A 5–8-mm margin was added in each plane to generate a planning target volume (PTV). RT was administered once daily in 30 fractions for 5 d per week, for a total dose of 60 Gy in 2.0 Gy fractions.

Patients received two cycles of concurrent chemotherapy with 75 mg/m^2^ of cisplatin administered on d 1 and 29, and 700 mg/m^2^ of 5-FU administered as a continuous intravenous infusion for 96 h on d 1–4 and 29–32.

### FDG PET/CT scanning

All patients underwent an initial PET/CT scan for tumor staging and therapy planning. A “failure” FDG PET/CT (failure PET/CT) was performed for patients with pathologically established treatment failure. FDG PET/CT scans were obtained with an advanced PET/CT scanner (Discovery LS; GE Healthcare, Waukesha, WI, USA). All patients fasted for at least 6 h before the examination, and blood glucose levels were recorded before injection of 5.50 MBq/kg of FDG. Images were acquired 60 min after injection. Scanning was performed in whole-body mode from head to thigh for 5 min per field of view, each covering 14.5 cm, at an axial sampling thickness of 4.25 mm per slice. Unenhanced CT scan was performed with an X-ray tube voltage peak of 120 kV, 90 mA, a 6:1 pitch, a slice thickness of 4.25 mm, and a rotational speed of 0.8 sec per rotation. Both PET and CT scans were performed with patients under normal shallow respiration. PET data sets were reconstructed iteratively using CT data for attenuation correction. PET, CT, and fused PET/CT images displayed as coronal, sagittal, and transaxial slices were viewed on the Philips extended brilliance workstation.

### Image analysis

SUVs were calculated using an elliptical region of interest (ROI) drawn around the area of increased uptake within the esophagus. The maximum SUV was recorded as the SUV_max_. MTV was defined as the volume of hyper-metabolic tissue with an SUV greater than a defined threshold of 2.5 [25, 26].

Using an automatic rigid registration algorithm based on CT scan information, failure PET/CT images were fused to initial PET/CT images on the Philips extended brilliance workstation. If the automatic registration showed a large deformation between the two CT scans, the images were manually registered on the surrounding anatomy of the tumor. On initial PET/CT images, the pre-subvolume was delineated using a relative threshold method (40%, 50%, 60%, and 70% of primary tumor SUV_max_) as Pre40%, Pre50%, Pre60%, and Pre70%, respectively. On failure PET/CT images, an SUV threshold of 2.5 was used to delineate LF volume. 90% SUV_max_ was used to delineate the failure region hotspot. The overlap fraction (OF) of the primary tumor was calculated as pre-subvolume ∩ failure subvolume / V_min_, where ∩ denotes the intersection, and V_min_ is the smaller of these two subvolumes [18].

### Follow-up

Patients were asked to visit the clinic within 60 d after completion of all therapies. Follow-up was performed until treatment failure every three months for the first year, every six months during the following three years, and annually thereafter. At each follow-up visit, patients underwent barium swallow, thoracic, and abdomen CT scan. Upon clinical or imaging signs of possible LF, patients underwent esophagogastroscopy and biopsy. LF included failure in the primary tumor. Regional failure included failure in regional lymph nodes. Distant failure included failure in any site beyond the primary tumor and regional lymph nodes. Further treatments were designed based on the patient's condition and included surgery, chemotherapy and/or radiotherapy.

### Statistical analysis

Normally distributed data were presented as means ± standard deviations and categorical data as proportions. OS and LC were estimated using the Kaplan-Meier method and analyzed using the log rank test. Patients were censored at the time of last follow-up. Univariate and multivariate Cox proportional hazards models were fit to evaluate potential associations between LC and clinical factors; results are presented as hazard ratios (HR) and 95% confidence intervals (95% CI). Receiver operating characteristics (ROC) analysis was used to assess the area under curve (AUC) and the optimal cut-off values for predicting failure in pre-treatment FDG high uptake regions. SPSS 17.0 (IBM, Armonk, NY, USA) software was used for statistical analysis. Two-sided P-values<0.05 were considered statistically significant.
